# Dietary Intake and Supplement Use in Competitive Women Bodybuilders

**DOI:** 10.3390/sports11080158

**Published:** 2023-08-18

**Authors:** John E. Haubenstricker, Jerry W. Lee, Gina Segovia-Siapco, Ernesto Medina

**Affiliations:** School of Public Health, Loma Linda University, Loma Linda, CA 92350, USA; jlee@llu.edu (J.W.L.); gsiapco@llu.edu (G.S.-S.); emedina@llu.edu (E.M.)

**Keywords:** women, bodybuilder, dietary supplement, diet quality, nutrition, protein, fat, carbohydrate, sport

## Abstract

(1) Background: Women bodybuilders use extreme diets, dietary supplementation, and training regimes to sculpt their physiques. Women’s participation in bodybuilding competitions has increased since the 1980s. Currently, studies on their dietary intake and supplement use are limited. Their dietary intake may be of poor quality and low in several micronutrients, while supplement use appears to be omnipresent. Thus, the aim of this study was to examine and compare the dietary intake, supplement use, and diet quality of in-season and off-season women bodybuilders. (2) Methods: In a cross-sectional design, we compared dietary intake, supplement use, and diet quality between seasons in women bodybuilders (*n* = 227). An online questionnaire was developed, validated, and administered to assess all non-dietary and supplement variables. The Automated Self-Administered 24 h Dietary Assessment Tool was used to collect four 24 h dietary recalls. The Healthy Eating Index-2015 (HEI-2015) was used to calculate diet quality. The analysis of covariance and Welch’s *t*-tests were used to assess the differences between in-season and off-season women bodybuilders’ dietary intake, supplement, and HEI-2015 variables. (3) Results: In-season competitors reported consuming significantly less energy, carbohydrates, and fat but more protein than off-season competitors. All competitors consumed excess protein, while in-season competitors consumed excess fat and off-season competitors consumed less energy than the physique athlete nutrition recommendations. All competitors’ micronutrient intakes were above the Dietary Reference Intakes. Supplements were used by all competitors, and the mean number used was similar between seasons. The HEI-2015 scores were not significantly different between seasons yet were below the US Dietary Guidelines for Americans. (4) Conclusion: Women bodybuilders would benefit from health education to achieve physique athlete nutrition recommendations, improve diet quality, and safe/efficacious supplement use to reach physique goals and improve overall health.

## 1. Introduction

Bodybuilding is a sport in which competitors are judged on their physical appearance, stage presence, and posing typified by the achievement of a muscular, defined, proportional, and symmetrical physique [1,2]. The precise mix of these physical characteristics is determined by the different competition divisions [2,3]. Since the breakthrough of female participation in the 1980s, there has been a dramatic increase in the number of women competing in bodybuilding, particularly in the newer divisions, i.e., bikini, figure, physique, and wellness [4,5,6,7]. To develop their division-specific physique, competitive women bodybuilders spend an extensive amount of time, e.g., months to years, implementing rigorous training, nutrition, and dietary supplement regimes in a phase of bodybuilding competition preparation called the “off-season” [8,9,10,11]. Upon completion of the off-season, competitive women bodybuilders transition into the next phase of preparation called the “in-season” [11,12]. Competitive women bodybuilders will spend 8–30 weeks manipulating their training, nutrition, and dietary supplements to reduce body fat and maintain the muscle accrued in the off-season to meet the division-specific physique requirements for competition [1,2,6,10,13,14,15]. Although there is considerable interindividual variability in the precise duration spent in these two phases, it has been estimated that they comprise of the majority of time (~95–99%) spent preparing for a bodybuilding competition [12].

A recent systematic review found eight studies assessing dietary and supplement intake in competitive women bodybuilders [10]. Heyward, et al. [16] indicated off-season competitors consumption to be, on average, 1636.2 kcals/day, 102 g/day of protein (1.7 g/kg/day), 208 g/day of carbohydrates (3.6 g/kg/day), and 42 g/day of fat (21% of energy). In three studies, in-season women bodybuilders consumed approximately 894–1542 kcals/day, ~48–131 g/day of protein (0.8–2.4 g/kg/day), ~160–199 g/day of carbohydrates (2.8–3.6 g/kg/day), and ~9–23 g/day of fat (9–17% of energy) [17,18,19]. This review, however, noted all the studies were dated as they were published in the 1980s and 1990s. Most of the studies did not include the energy or macro- and micronutrient contribution of dietary supplements or alcohol, likely underestimating actual intake. In addition, many of these studies did not include a comprehensive examination of their micronutrient intake and failed to indicate whether micronutrient intake exceeded the tolerable upper intake level (UL) [10]. Finally, the caliber of women bodybuilders was not well described in these studies, nor was the season of training. In totality, most of the literature on competitive women bodybuilders assessed in this systematic review is dated, limited, or poor quality.

Since this systematic review, 11 studies have explored dietary intake in competitive women bodybuilders. Two case studies examining off-season competitive women bodybuilders indicated these competitors consume, on average, 2010–2500 kcals per day, ~120–144 g of protein per day (2.1–2.2 g/kg/day), ~225–331 g of carbohydrates per day (4.1–4.9 g/kg/day), and ~67–70 g of fat per day (1.0–1.28 g/kg/day) in the off-season [7,20]. During the in-season, women bodybuilders consume, on average, 965–2358 kcals/day, 126.1–204 g of protein per day (2.0–3.5 g/kg/day), 20–260 g of carbohydrates per day (0.33–3.9 g/kg/day), and 20–52 g of fat per day (0.3–0.8 g/kg/day) [6,7,15,20,21,22,23].

Although these studies are current, most still have limitations like the earlier research. None of the four case studies examined micronutrient intake, alcohol’s contribution to energy intake, nor did they include all dietary supplements in the analyses [6,7,20,23]. Only two studies included the contribution of all dietary supplements to energy and macronutrient intake, but not to micronutrient intake [15,21]. Those same two studies reported that United Kingdom (UK) competitive women bodybuilders, on average, used 5.4 [most commonly used supplements = protein powder, multivitamin, branch chain amino acids, creatine, omega 3 fatty acids, and fat burners] and 8.8 [most commonly used supplements = protein powders, branch chain amino acids, vitamin C, omega 3 fatty acids, multivitamins, and creatine] dietary supplements during in-season, respectively [15,21]. Tinsley et al. [6] indicated a United States (US) figure competitor used 9 to 21 dietary supplements during in-season. It appears that dietary supplement use among competitive women bodybuilders may be higher in the US, potentially affecting overall dietary intake to a greater extent [6,15,21]. Ismael et al. [22] found that competitive women bodybuilders were below the Recommended Dietary Allowance/Adequate Intake (RDA/AI) levels for vitamin A, vitamin D, vitamin E, iron, potassium, and fiber. Although none of the micronutrients were over the UL, the contribution of dietary supplements was also not included in the dietary analysis. There continues to be a paucity of research on competitive women bodybuilders, particularly involving the examination of micronutrient intake and the inclusion of dietary supplements and alcohol to overall dietary intake.

Although the literature on dietary intake in competitive women bodybuilders is lacking, it appears that their dietary intake may be suboptimal for health [10,22]. Competitive women bodybuilders adhere to a strict diet during the in-season, often following the same dietary plan for weeks [15]. Several studies have reported on the types of foods consumed by in-season competitive women bodybuilders, often citing high intake of lean protein sources, e.g., chicken breast, egg whites, lean fish, canned tuna in water, and low intake of vegetables, fruits, and whole grains, with the avoidance of dairy, legumes, red meat, whole eggs, alcohol, fats, and oils [17,24,25]. Two recent studies on UK in-season competitive women bodybuilders have found similar food group patterns as their US counterparts, but have a higher intake of vegetables, cereals, nuts, and dairy [15,21]. The high dairy intake is due to the inclusion of dairy-based protein powders in the diet. Although off-season competitive women bodybuilders’ dietary intake varies more, dietary intake patterns like those of the in-season remain [17,26]. Off-season competitive women bodybuilders consistently consume lean protein sources (skinless chicken breasts, tuna canned in water, egg whites, and lean beef), limited vegetables (dried beans, starchy vegetables, broccoli, and lettuce), but slightly increased low-fat dairy (low-fat and skim milk products) and fat intakes (whole eggs, cookies, ice cream, and cake) than in-season competitive women bodybuilders [17,26].

In the only study that examined nutrient density, a measure of diet quality, in in-season and off-season competitive women bodybuilders, nutrient density values for protein and several micronutrients were compared to the Index of Nutritional Quality [26]. The Index of Nutritional Quality is a ratio of an RDA of a nutrient divided by the energy intake recommendation for a person’s age multiplied by 1000 kcals [27]. All nutrient density values in both in-season and off-season competitors exceeded the RDA/AI for all nutrients, except calcium, zinc, and iron. The low intakes of calcium, zinc, and iron were possibly due to the low zinc and iron content of protein sources and a low whole grain and dairy intake. The exclusion of dairy foods, the low intake of whole grains, and a low variety in protein sources has the potential to limit the intake of several essential micronutrients in competitive women bodybuilders.

Competitive women bodybuilders’ diet quality may be subpar for the rigors of training, while increasing their risk for morbidity and mortality [28,29]. In addition, the consumption of excess protein may compromise adequate carbohydrate and essential fatty acid intake, thus impairing performance and anabolism, especially in the off-season [29]. One valid and reliable tool for assessing diet quality and conformance with the Dietary Guidelines for Americans is the Healthy Eating Index-2015 (HEI-2015) [30]. The HEI-2015 encompasses key recommendations of the 2015 Dietary Guidelines for Americans and is used to evaluate nutrition recall and survey data to assess dietary patterns, diet quality, and certain areas of the food environment [31]. The HEI-2015 is also used by the Center for Nutrition Policy and Promotion to monitor diet quality in the US and associations between diet quality and health outcomes [32]. The HEI-2015 has also been used extensively in the literature and can be used to compare diet quality among different athletes [33,34]. To our knowledge, the HEI-2015 measure of diet quality has not been assessed in competitive women bodybuilders.

Research examining dietary intake, dietary supplement intake, and diet quality in competitive women bodybuilders is lacking, particularly using validated measurement tools. Much of the research fails to include the contribution of supplements and alcohol to overall dietary intake and a thorough examination of micronutrient intake. Almost absent in the literature is the examination of dietary intake and dietary supplement intake in off-season bodybuilders; the examination of dietary intake in athletes competing in the new women’s bodybuilding divisions, i.e., bikini, physique, and wellness; and the overall diet quality of competitive women bodybuilders. The diet rigidity and large protein intakes displayed by competitive women bodybuilders may impair diet quality [26] and hinder performance, particularly if carbohydrates are displaced, during the off-season [29]. In addition, the ubiquitous consumption of dietary supplements [6,15,21] may increase the potential for long-term, negative health consequences [35]. Thus, the primary aim of the current descriptive study is to examine the dietary intake of in-season and off-season competitive women bodybuilders. The secondary aims were to examine dietary supplement use and diet quality of in-season and off-season competitive women bodybuilders.

## 2. Materials and Methods

### 2.1. Design

**Figure 1:**
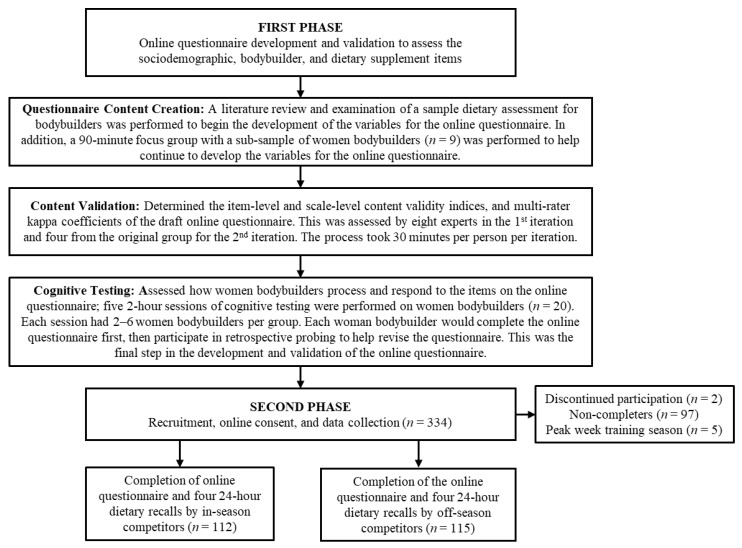
A flowchart overview of the study.

The study employed a cross-sectional design, which is where the “description of [a] population is made through a unique temporal point” [36], to compare dietary intake, dietary supplement use, and diet quality between in-season and off-season competitive women bodybuilders. The study had two main phases. The first phase was the development of an online questionnaire to assess dietary supplement, sociodemographic, and bodybuilder variables. The second phase was the recruitment of participants, the administration of the online questionnaire via Qualtrics [37], and the collection of four 24 h dietary recalls via Automated Self-Administered 24-Hour (ASA24) Dietary Assessment Tool, version 2020, developed by the National Cancer Institute, Bethesda, MD [38]. Figure 1 shows the overview of the study.

### 2.2. Participants

The women bodybuilder sample size was based on the power analysis from our separate study examining dietary behaviors in only in-season competitive women bodybuilders (*n* = 112); thus, we endeavored to recruit a similar number of off-season competitive women bodybuilders (*n* = 115) for comparison [39]. Competitive women bodybuilders were recruited via purposive and snowball sampling from bodybuilding forums (e.g., bodybuilding.com [accessed 3 August 2020], t-nation.com [accessed 3 August 2020]), social media platforms (e.g., Instagram and Facebook), known physique coaches, industry colleagues, local gyms, and known competitive women bodybuilders throughout the US. To participate in the study, eligible participants had to be fluent in English, at least 18 years old and female, plus reside in the US with internet access. Additionally, participants must be currently competing, planning on competing next year, or have competed in the past year in one of the physique divisions, i.e., bodybuilding, bikini, figure, fitness, physique, or wellness. Interested participants were sent a link to the study website to complete an online screener to see if they met the inclusion criteria to participate in the study. Participants who qualified to participate in the study received a link after the online screener to the Qualtrics questionnaire. To reduce the chance a participant would miss answering a question, each section of the questionnaire had a response request window appear requiring participants to select “continue without answering” or “answer the question” before they could move onto the next section. Participants who completed the questionnaire were emailed or texted a unique username and password to complete the four dietary recalls via ASA24. Text and email reminders were sent out once a week to remind participants to complete their dietary recalls. Once both the questionnaire and four dietary recalls were completed, participants were compensated for their time, i.e., a total of 2–3 h, with a USD 100 incentive. All participants provided consent online and a copy of this consent prior to beginning the study and completed the study between July 2020 and November 2020. Additionally, participants were provided with the investigator’s contact information if questions arose during consent or any part of the study. Participants were also provided with Loma Linda University’s patient relations contact information for information and assistance with complaints or concerns about this study. Data used for the analyses were deidentified and saved in password-protected files. Raw data from all participants were saved in a password-protected and encrypted external hard drive that was kept in a locked safe in the investigator’s office. The study was performed in accordance with the Declaration of Helsinki, and all protocols, marketing materials, and study website were approved by the Loma Linda University Health Institutional Review Board (IRB# 5180399). This study is covered by a Certificate of Confidentiality from the National Institutes of Health (#CC-OD-20-527).

### 2.3. Definitions

Several definitions are proffered to enhance clarity and understanding of the competitive women bodybuilders’ training phases, which include the following:

- In-season—the period in a competitive bodybuilder’s training cycle dedicated to preparing for a competition, which usually involves a reduction in energy intake, i.e., called cutting by bodybuilders, and an increase in cardiovascular exercise to obtain very low levels of body fat in order to expose the underlying muscle to the degree required for the competitor division, i.e., bodybuilder, bikini, figure, fitness, physique, and wellness [13].
- Off-season—the period in a competitive bodybuilder’s training cycle that is dedicated to recovery, repair, and physique improvements via resistance exercise to enhance muscular hypertrophy, low level/absent cardiovascular exercise, and increased energy intake, i.e., called bulking by bodybuilders [8].
- Peak week—this is the last week of the in-season training phase before competing on stage. During this period, competitive women bodybuilders may manipulate their fluids, electrolytes, and carbohydrates, e.g., carbohydrate loading, concomitantly with a reduction in training in an attempt to display their best physique on stage, e.g., enhanced muscle size and definition [1].

### 2.4. Variables

#### 2.4.1. Dietary Intake and Dietary Supplements

An average of four, non-consecutive 24 h dietary recalls (three weekdays and one weekend day) were collected and analyzed using the ASA24 [38]. Participants were instructed to report all food, fluids, and dietary supplements they consumed during the previous day from midnight to 11:59 pm. Participants were provided the English version of the *Participant Quick Start Guide for 24-Hour Recall using ASA24-2018 & ASA24-2020* instructional materials to assist in completing the dietary recalls [40]. Participants also had technical support available via email or text when completing the dietary recalls. Data were reviewed and cleaned, and known issues were addressed based on the National Cancer Institute’s recommendations by a registered dietitian [41,42]. The total intake of energy, macronutrients, micronutrients, and other nutrients, i.e., fiber, sugar, caffeine, cholesterol, saturated fat, monounsaturated fat, polyunsaturated fat, linoleic acid, and α-linolenic acid, were calculated for both in-season and off-season competitive women bodybuilders.

Total dietary supplement use, types of dietary supplements, and training phase of use during the past 12 months were assessed via the self-administration of the online questionnaire [37]. Over 60 individual dietary supplements with an option for “other dietary supplements” were assessed in the questionnaire. The contribution of dietary supplements to energy intake, macronutrients, micronutrients, and other nutrients was also included in the analyses.

#### 2.4.2. Diet Quality

The HEI-2015 was used to calculate diet quality and the 13 component scores included nine diet adequacy components, i.e., total fruit, whole fruit, total vegetables, greens and beans, whole grains, dairy, total protein foods, seafood and plant proteins, and fatty acids, and four moderation components, i.e., refined grains, sodium, added sugars and saturated fats [31]. Dietary supplements are not included in the calculation of the HEI-2015 scores. A detailed explanation of the HEI-2015 components, their standards, and computation has been described previously [31]. Briefly, the total component score was adjusted for energy intake per 1000 calories or as a percent of calories [31]. The maximum value of all the component scores equals 100, and a grading system has been developed to delineate diet quality scores: F (0–59), D (60–69), C (70–79), B (80–89) and A (90–100) [31]. A higher score in any of the nine diet adequacy components and moderation components equates to better diet quality [31].

#### 2.4.3. Sociodemographic and Bodybuilder Variables

The online questionnaire developed for this study included items to collect the following demographic information: age, race, ethnicity, exercises as part of a competitor’s training protocol performed for at least 10 min over past 7 days, height, current weight, educational attainment, employment status, and household income from all sources. The exercises as part of a competitor’s training protocol items were developed based on the past-week Modifiable Activity Questionnaire adjusted for the specific exercises routinely performed by competitive women bodybuilders [43]. Exercises and associated metabolic equivalent (MET) values were obtained from the 2011 Compendium of Physical Activities to calculate total MET minutes per week, resistance training MET minutes per week, and aerobic training MET minutes per week [44]. Educational attainment, race, and ethnicity items were based on the 2020 Census [45,46]. Employment status and household income from all sources items were adapted from the Behavioral Risk Factor Surveillance System 2020 questionnaire [47].

The online questionnaire also included a section with the following items: bodybuilding divisions (i.e., bodybuilding, bikini, fitness, figure, physique, and wellness), training phase (i.e., in-season, off-season, or peak week), competition status (i.e., amateur and professional), total number of years of competition, most recent top competition placing details (i.e., organization name, year, placing, professional/amateur competition, and whether professional status was awarded), competitor type (e.g., designated natural vs. all others), most recent competition weight, lowest off-season weight since the last competition, highest off-season weight since last the competition, total number of years competing, total number of competitions, time in weeks since the last competition, and time remaining till the next competition in weeks (in-season competitors only).

### 2.5. Online Questionnaire Development and Validation

There were three main steps to the development and validation of the 549-item online questionnaire used to assess dietary supplement, sociodemographic, and bodybuilder variables in competitive women bodybuilders. The three steps include (a) questionnaire content creation, (b) content validation, and (c) cognitive testing. After those steps were completed, but prior to the collection of the four 24 h dietary recalls, the questionnaire was administered online via Qualtrics.

#### 2.5.1. Questionnaire Content Creation

Items for the online questionnaire on dietary supplement, sociodemographic, and bodybuilder variables were created based on the limitations discussed in a systematic review [10], items from a sample dietary assessment questionnaire for bodybuilders [21], and the results from a focus group of a representative sub-sample of the study. The purpose of the focus group was to identify and provide insight into the relevant dietary supplement, sociodemographic, and bodybuilder characteristics in competitive women bodybuilders. A 90 min in-person focus group was conducted using a sub-sample of nine competitive women bodybuilders, i.e., bikini, figure, and physique competitors, who met the inclusion criteria for the study. Each participant was compensated for her time with a USD 30 incentive. A detailed explanation of the focus group methodology and analyses has been described previously [48].

#### 2.5.2. Content Validation

Content validity for the draft version of the final questionnaire was assessed using item-level and scale-level content validity indices (CVIs) and the multi-rater kappa coefficient as discussed in Polit et al. [49]. Experts were chosen based on the established criteria [50], which included professionals with subject matter expertise in sports nutrition, eating behavior, and exercise physiology, i.e., sports dietitians, from across the US. These experts were recruited via purposive and snowball sampling from known dietetic professionals and colleagues. Experts determined the content validity of the questionnaire through evaluating the relevance and clarity of each item, providing suggestions to enhance clarity, and assessing the comprehensiveness of the entire questionnaire. The entire process took approximately 30 min per participating expert. Participants were provided with the website link to the online questionnaire and, upon completion, were compensated for their time with a USD 40 incentive. Content validation required two iterations with the first expert panel of eight to assess the initial item-level CVI and provide feedback to improve the questions and answers. A second expert panel of four were chosen from the same initial group based on recommendations from Polit et al. [49] to evaluate the item-level CVI, the scale-level CVI, and provide feedback and comments for the revised questionnaire.

For the calculation of the item-level CVI, each rater/expert rated each item for its relevance to the underlying construct using a four-point ordinal scale (not relevant, somewhat relevant, quite relevant, and highly relevant). This was then collapsed into a binary scale (1 = quite relevant, highly relevant vs. 0 = not relevant, somewhat relevant). Item-level CVI for each item was calculated as the proportion of raters/experts rating the item as relevant on this binary scale.

To adjust for inter-rater agreement by chance, the modified multi-rater kappa (measure of agreement among the raters/experts that the item is relevant, over and above that expected by chance) was calculated for each item using the following formula:

$$modified\;kappa=\frac{item\;CVI-\left[\frac{N!}{A!\left(N-A\right)!}\right].5N}{1-\left[\frac{N!}{A!\left(N-A\right)!}\right].5N},$$

where $\left[\frac{N!}{A!\left(N-A\right)!}\right].5N=the\;probability\;of\;chance\;agreement$ with N = the number of raters/experts and A = the number of raters/experts rating the item as relevant. Previously described guidelines indicate the following evaluation criteria for the modified kappa: (a) fair = 0.40–0.59, (b) good = 0.60–0.74, (c) excellent ≥ 0.74 [51,52].

The scale-level CVI was calculated as the average of all I-CVIs for items in a given construct, e.g., sociodemographic or bodybuilder characteristics, by all raters/experts [49]. The initial iteration of the questionnaire revealed 15 out of 101 questions with an item-level CVI less than 0.78; thus, additional revisions were needed to ensure a scale-level CVI of 0.90 or greater and achieve a modified kappa rating of good to excellent as recommended by Polit et al. [49]. The second iteration revealed a scale-level CVI of 0.92 as the lowest value for any of the scales and a modified kappa rating of excellent for all scales.

#### 2.5.3. Cognitive Testing

After content validity was established, the questionnaire was pre-tested using the cognitive testing technique of retrospective probing [53,54]. This was performed to study the way competitive women bodybuilders process and respond to the items in the online questionnaire to reduce measurement error and improve the quality of data [55,56]. First, competitive women bodybuilders completed the questionnaire online and uninterrupted to allow the interviewer the ability to observe any technical difficulties, while also evaluating their ability to answer and navigate the online questionnaire unaided [54]. Next, respective probing was conducted by the interviewer as an in-depth, semi-structured interview using probing questions to explore question comprehension (“Please tell me in your own words what X question was asking for?”), retrieval (“What X question was difficult to answer, i.e., questions which you had a difficult time remembering the information needed to answer the question?”), response (“What X question do feel you could not find the answer you wanted from the provided answer choices?”), and general (“What X question was difficult to answer?”) processes [53]. After each session, the researcher’s notes and audio recording transcription of the retrospective probing question’s responses were aggregated and summarized for each question to revise the questionnaire [54]. The revised questionnaire was compared to the original version to demonstrate the revision had either fewer problems or eliminated problems within the questionnaire [55]. Retrospective probing was conducted with a different set of 2–6 competitive women bodybuilders, i.e., bikini, figure, physique, and wellness competitors, at each 2 h cognitive testing session on five separate occasions. This required a total of 20 competitive women bodybuilders to detect at least 50% of the more serious problems affecting measurement error 50–90% of the time for our questionnaire [57] to achieve saturation without new high-impact problems [58]. Participants who completed the cognitive testing were compensated for their time with a USD 60 incentive.

### 2.6. Data Storage and Analyses

The demographic and dietary supplement intake data were collected with Qualtrics and secured on their website. The dietary intake and diet quality data were collected with ASA24 by the National Cancer Institute and secured on their website. Both websites offer password protection, while the National Cancer Institute also has a separate researcher site to assess the dietary data [59]. Data culled from each website for the analyses was secured on a password-protected and encrypted external hard drive that is kept in a locked safe in the investigator’s office. In the case of missing data or an unusual value being detected, the researcher attempted to contact the participant to rectify the issue. During the analyses, missing data were handled using multiple-imputation via the EM algorithm as described by Graham [60].

The IBM SPSS Statistics Version 28.0 (IBM Corp, Chicago, IL, USA) was used to perform all statistical analyses. All data are presented as mean and 95% confidence interval unless otherwise stated. The HEI-2015 score per participant and group level score was calculated using SAS. A detailed explanation of HEI-2015 methodology, calculations, and interpretation has been described previously [31,61]. Pearson correlations were used to evaluate all sociodemographic, training, and competition characteristics for confounding with the dietary intake, dietary supplement use, and diet quality variables. All significant confounding variables were controlled for in the final analyses for these dietary variables and described next in this paragraph. Differences between in-season and off-season competitive women bodybuilders’ sociodemographic and bodybuilder continuous variables were 90% winsorized and assessed using analysis of covariance (ANCOVA) and Welch’s *t*-tests, controlling for current weight and total activity [62,63]. Pearson Chi-Square or Fisher–Freeman–Halton exact tests, controlled for current weight and total activity, were used to assess the differences between in-season and off-season competitive women bodybuilders’ sociodemographic and bodybuilder categorical variables. The ANCOVA and Welch’s *t*-tests, controlled for current weight, total activity, the number of days required to complete the four 24 h dietary recalls, and the categorical variable completion of non-consecutive dietary recalls (yes or no) were used to assess the differences between in-season and off-season competitive women bodybuilders’ dietary intake, dietary supplements, and HEI-2015 variables. All continuous variables were 90% winsorized [62]. Benjamini and Hochberg multiple comparison testing was employed for all dietary intake and HEI-2015 analyses [64,65]. The Sidak multiple comparison testing was employed for dietary supplement use. Significance was set a priori at *p* < 0.05.

## 3. Results

### 3.1. Participant, Sociodemographic, Training, and Competition Characteristics

A total of 334 participants enrolled in the study, with 2 participants requesting to discontinue participation, 3 participants residing outside the US, and 97 who did not complete all four 24 h dietary recalls. Another five participants were omitted from the analyses due to being in the peak week training season. This left a total of 227 participants (68%) who met the inclusion criteria and completed the entire study. After completion of the questionnaire, the average number of days participants took to complete the four 24 h dietary recalls was 13.0 days (95% CI 12.2–13.8). The time to complete the dietary recalls was not statistically different (*p* = 0.286) between in-season (mean = 12.6 days, 95% CI 11.5–13.7) and off-season competitors (mean = 13.4 days, 95% CI 12.3–14.5). Sociodemographic, training, and competition characteristics are presented in Table 1 and Table 2. The mean total activity for in-season competitive women bodybuilders (mean = 4829.2 MET min/wk, 95% CI 4411.6–5246.7) was significantly greater (*p* = 0.001) than the off-season competitors (mean = 3935.3 MET min/wk, 95% CI 3577.5–4293.1). Specifically, the mean aerobic training for in-season competitors (mean = 2658.2 MET min/wk, 95% CI 2322.1–2994.3) was significantly greater (*p* < 0.001) than for the off-season (mean = 1880 MET min/wk, 95% CI 1582.5–2177.5). Resistance training, however, was not significantly different between groups (in-season mean = 1896.2 MET min/wk, 95% CI 1709.3–2083.2; offseason mean = 1788.1 MET min/wk, 95% CI 1597.3–1978.8). The current weight for in-season competitors (mean = 58.6, 95% CI 57.4–59.9) was significantly less (*p* < 0.001) than off-season competitors (mean = 62.6, 95% CI 61.2–63.9). There were more non-Hispanic/Latino/Spanish individuals in the in-season group (*n* = 102, 92%) than the off-season group (*n* = 95, 82.6%). However, after adjustment for the number of significance tests carried out, this difference did not remain significant. There were no other significant differences between competitors for any other sociodemographic or competition characteristics.

### 3.2. Dietary Intake

#### 3.2.1. Energy and Macronutrients

The mean absolute and relative energy, macronutrient, i.e., protein, carbohydrate, and fat, and mean relative energy (kcal/kg) and macronutrient intakes (g/kg) are presented in Table 3 and Table 4, respectively. In-season competitors’ mean absolute and relative energy (mean = 1779.8 kcal, 95% CI 1689.2–1870.4; mean = 33.3 kcal/kg, 95% CI 31.6–34.9) and carbohydrate intakes (mean = 156.0 g, 95% CI 143.5–168.5; mean = 2.9 g/kg, 95% CI 2.7–3.1) were significantly less (*p* < 0.001) than off-season competitors’ mean absolute and relative energy (mean = 2032.9 kcal, 95% CI 1940.6–2125.1; mean = 38.0 kcal/kg, 95% CI 36.3–39.7) and carbohydrate intakes (199.1 g, 95% CI 186.3–211.8; mean = 3.7 g/kg, 95% CI 3.5–4.0). In-season competitors’ mean absolute fat intake (mean = 60.1 g, 95% CI 55.6–64.6) and relative fat intake (mean = 1.1 g/kg, 95% CI 1.0–1.2) were significantly less (*p* ≤ 0.001) than off-season competitors’ mean absolute fat intake (mean = 70.9 g, 95% CI 66.3–75.5) and relative fat intake (mean = 1.3 g/kg, 95% CI 1.2–1.4). Absolute (mean = 155.5 g, 95% CI 148.5–162.5) and relative protein intake (mean = 2.9 g/kg, 95% CI 2.8–3.0) for in-season competitors was significantly greater (*p* = 0.005) than off-season competitors’ absolute (mean = 141.2 g, 95% CI 134.1–148.3) and relative protein intake (mean = 2.6 g/kg, 95% CI 2.5–2.8). After 90% winsorization of the alcohol data, mean values were zero for all competitors. Only 3.5% of the entire cohort (*n* = 33) consumed alcohol, with in-season (1.8%; *n* = 8) and off-season (5.2%; *n* = 25) mean alcohol intakes being 0.02 g (95% CI 0.01–0.03) and 0.05 g (95% CI 0.03–0.07), respectively.

The mean relative energy (kcal/kg) and macronutrient intakes (g/kg) compared to the nutrition recommendations are presented in Table 4. The relative mean energy (38.0 kcal/kg, 95% CI 36.3–39.7), carbohydrate (3.7 g/kg, 95% CI 3.5–4.0), and fat (1.3 g/kg, 95% CI 1.2–1.4) intakes for off-season competitors met 100% of the nutrition recommendations. The relative mean carbohydrate intake (2.9 g/kg, 95% CI 2.7–3.1) for in-season competitors met 100% of the nutrition recommendations. Protein intake for both in-season (2.9 g/kg, 95% CI 2.8–3.0) and off-season (2.6 g/kg, 95% CI 2.5–2.8) competitors and fat intake (1.1 g/kg; 95% CI 1.0–1.2) only for in-season competitors exceeded the nutrition recommendations.

#### 3.2.2. Micronutrients and Other Nutrients

The mean micronutrient and other nutrient intakes are presented in Table 5. The in-season competitors’ mean sugar (mean = 48.0 g, 95% CI 43.1–52.9, *p* < 0.001), saturated fat (16.1 g, 95% CI 14.5–17.7, *p* < 0.001), monounsaturated fat (21.6 g, 95% CI 19.8–23.5, *p* = 0.007), polyunsaturated fat (13.1 g, 95% CI 11.9–14.3, *p* = 0.004), and linoleic acid (10.6 g, 9.6–11.7, *p* < 0.001) intakes were significantly less than off-season competitors’ mean sugar (62.0 g, 95% CI 57.0–66.9), saturated fat (20.2 g, 95% CI 18.6–21.8), monounsaturated fat (25.3 g, 95% CI 23.4–27.2), polyunsaturated fat (15.5 g, 95% CI 14.4–16.7), and linoleic acid (13.3 g, 95% CI 12.3–14.3) intakes. The in-season competitors’ mean vitamin A intake (1544.7 µg_RAE, 95% CI 1355.0–1734.5) was significantly greater (*p* = 0.001) than off-season competitors’ (1090.8 µg_RAE, 95% CI 897.7–1283.9). There were no other significant differences between groups for any of the other micronutrients or other nutrients.

The in-season and off-season competitors’ micronutrient intakes were compared to the RDA/AI reference values (Supplementary Materials, Table S1). In-season competitors’ linoleic acid intake (10.6 g) was below the RDA/AI (88.6%). Similarly, off-season competitors’ fiber intake (24.4 g) was below the RDA/AI (97.6%). All other micronutrient intakes for both groups were greater than the RDA/AI reference values. Magnesium (in-season = 537.4 mg, off-season = 515.4 mg) and niacin (in-season = 51.8 mg, off-season = 48.3 mg) intakes were above the UL (magnesium = 350 mg/day, niacin = 35 mg/day) in both groups. All other micronutrient intakes for both groups were below the UL.

### 3.3. Dietary Supplements

Dietary supplements used over the past 12 months are presented in Table 6. All competitive women bodybuilders reported using dietary supplements. In-season competitors used significantly (*p* = 0.030) more dietary supplements during the in-season training season (mean = 11.2) than off-season competitors (mean = 9.5), while off-season competitors (mean = 10.3) used significantly (*p* = 0.034) more dietary supplements than in-season competitors (mean = 8.8) during the off-season training season. There were no significant differences between groups for total or peak week dietary supplement use. The mean in-season (mean = 11.2), off-season (mean = 8.8), and peak week (mean = 3.2) dietary supplement use within in-season competitors significantly (*p* < 0.05) decreased from in-season to off-season to peak week. The mean in-season (mean = 9.5) and off-season (mean = 10.3) dietary supplement use was significantly (*p* < 0.05) greater than the peak week (mean = 3.0) dietary supplement use within off-season competitors and both groups combined.

The frequency and percentage of all dietary supplements used compared across seasons during the past 12 months are shown in the Supplementary Materials, Table S2. A total of 60 types and groups of dietary supplements, including a miscellaneous dietary supplement category, were used at least once during one of the three seasons, i.e., in-season, off-season, or peak week. The dietary supplements that were used by more than 50% of the competitors across all training seasons were whey protein powder, multivitamins, creatine, energy drinks, fish oil, branched-chain amino acids, probiotics, and vitamin D. A total of 13 dietary supplements were not exclusively used across all seasons, with 4 dietary supplements only used during one season. In addition, several types of dietary supplements included in the miscellaneous dietary supplement category had a frequency of five or more: plant-based proteins (*n* = 29), preworkouts (*n* = 24), ashwagandha (*n* = 16), turmeric (*n* = 15), fat burners (*n* = 12), dandelion root (*n* = 7), green and red powders (*n* = 7), and biotin (*n* = 5).

The frequency, percentage, and overall position of dietary supplements shared among the seasons are presented in the Supplementary Materials, Table S3. A total of 41 dietary supplements (66.1%) were used across all three seasons, and 24 dietary supplements either increased or decreased at least five positions or more across the seasons. All other dietary supplements used across the training seasons changed less than five positions. The top five dietary supplements used during off-season (whey protein powder, multivitamins, creatine, energy drinks, and fish oils), in-season (multivitamins, whey protein powder, creatine, energy drinks, vitamin D), and peak week (multivitamins, vitamin D, vitamin C, creatine, and probiotics) were similar.

The number of dietary supplements divided into five distinct ranges of use are displayed with associated cumulative frequencies and percentages in the Supplementary Materials, Table S4. Competitors used between 1 and 34 dietary supplements over the past 12 months. Also, over 58% of competitors used between 6 and 15 dietary supplements, while over 31% of competitors used 16–34 dietary supplements over the past 12 months.

### 3.4. Diet Quality

Total diet quality and the 13 dietary component scores measured using the HEI-2015 for in-season and off-season competitors are presented in Table 7. The total vegetables score for in-season competitors (mean = 4.7, 95% CI 4.3–5.1) was significantly greater (*p* = 0.003) than the score for off-season competitors (mean = 3.9, 95% CI 3.5–4.3). Similarly, the added sugars score for in-season competitors (mean = 9.8, 95% CI 9.6–9.9) was significantly greater (*p* = 0.005) than the score for off-season competitors (mean = 9.5, 95% CI 9.3–9.6). There were no other significant differences between groups for total dietary quality or any of the other component scores.

## 4. Discussion

To the authors’ knowledge, this is one of the largest and most comprehensive descriptive dietary intake and supplement studies conducted to date on competitive women bodybuilders in the US. Also noteworthy, this study includes the newer women’s divisions, i.e., bikini, physique, and wellness, and the contribution of dietary supplement intake to energy, macronutrient and micronutrient intake during the in-season and off-season. Finally, to the authors’ knowledge, this is the only study to assess diet quality in this population. The study’s aims were to examine the dietary intake, dietary supplement use, and diet quality in in-season and off-season competitive women bodybuilders.

It is worth noting that, as presented in Table 2, 26.4% (*n* = 60) of competitive women bodybuilders indicated they have not competed, with about equal numbers identifying as in-season (*n* = 28) or off-season (*n* = 32). Given this, we examined if in-season and off-season competitive women bodybuilders who have competed and those who have not differ in dietary intake, dietary supplement use, and diet quality. For nutrient intakes, the mean caffeine (mean = 170.6 mg, 95% CI 135.3–205.9, *p* = 0.01), niacin (60.6 mg, 95% CI 53.5–67.7, *p* = 0.003), vitamin A (1818.8 µg_RAE, 95% CI 1575.0–2062.6, *p* < 0.001), and vitamin D (23.8 µg, 95% CI 17.2–30.5, *p* = 0.049) intakes for in-season competitors who have competed were significantly more than the mean caffeine (97.0 mg, 95% CI 53.8–140.3), niacin (43.7 mg, 95% CI 35.0–52.4), vitamin A (1056.0 µg_RAE, 95% CI 757.3–1354.7), and vitamin D (13.2 µg, 95% CI 5.1–21.4) intakes for in-season competitors who have not competed. Conversely, the mean caffeine (222.63 mg, 95% CI 143.9–300.6, *p* = 0.005) and vitamin D (37.0 µg, 95% CI 22.2–51.9, *p* < 0.001) intakes for off-season competitors who have not competed were significantly more than the mean caffeine (90.7 mg, 95% CI 21.7–159.6) and vitamin D (7.0 µg, 95% CI −6.1–20.0) intakes for off-season competitors who have competed. In-season competitors who have not competed had mean sugar (55.4 g, 95% CI 48.9–61.9, *p* = 0.007), fiber (27.7 g, 95% CI 24.6–30.8, *p* = 0.03), and α-Linolenic acid (1.6 g, 95% CI 1.4–1.8, *p* = 0.006) intakes which were significantly more than the sugar (43.9 g, 95% CI 38.6–49.2), fiber (23.3 g, 95% CI 20.8.3–25.8), and α-Linolenic acid (1.2 g, 95% CI 1.0–1.4) intakes of in-season competitors who have competed. Off-season competitors who have competed had a mean potassium intake (4236.3 mg, 95% CI 3711.0–4761.6, *p* = 0.02) which was significantly more than that of off-season competitors who have not competed (3441.6 mg, 95% CI 2844.8–4038.5). For diet quality, the saturated fats mean score (9.9, 95% CI 8.5–11.3, *p* = 0.03) for in-season competitors who have not competed was significantly greater than the saturated fats score of in-season competitors who have competed (7.9, 95% CI 6.7–9.1). Off-season competitors who have not competed had an added sugars mean score (10.0, 95% CI 9.7–10.3, *p* = 0.03) which was significantly greater than the added sugars score of in-season competitors who have competed (9.5, 95% CI 9.2–9.8). There were no other significant differences between the in-season and off-season groups for energy, all macronutrients, diet quality, dietary supplements, or micronutrients or other nutrient intakes. We found limited evidence that competitive women bodybuilders who have competed or have not competed differ in their dietary intake, diet quality, and supplement intakes.

### 4.1. Dietary Intake

#### 4.1.1. Energy and Macronutrients

In-season competitors’ mean absolute and relative energy, carbohydrate, and fat intakes were less than off-season competitors; however, protein intake was higher for in-season competitors than those of off-season competitors. Compared to the systematic review by Spendlove et al. [10], the absolute and relative energy, protein, and fat intakes for in-season and off-season competitive women bodybuilders in our study, presented in Table 3 and Table 4, were greater, except for carbohydrates. The greater absolute and relative values for energy, protein, and fat in the present study are possibly due to the inclusion of dietary supplements, particularly protein supplements, in our analyses, which were largely absent in the studies examined in the systematic review by Spendlove et al. [10]. In addition, the energy and macronutrient intakes may also depend on the dietary intake data collection methods and the exact time period during a competitors’ training season, particularly the in-season, when the data were ascertained [10].

Overall, the mean intakes for off-season and in-season competitors in the present study are like those in other recent studies [6,7,15,20,21,22,23]. The off-season competitors’ mean energy, protein, and fat intake values in the two case studies [7,20] were like those found for off-season competitors in the present study, except for a higher carbohydrate intake in those two case studies. Aside from the two case studies, to our knowledge, this is the first study since the late 1980s to examine dietary intake in off-season competitive women bodybuilders.

The mean energy, protein, carbohydrate, and fat intakes for in-season competitors from the current study were also like recent studies’ mean energy, protein, carbohydrate, and fat intake values [15,21,22]. Interestingly, when comparing our in-season competitors’ energy, carbohydrate, and fat intakes to the those of the four case studies [6,7,20,23], our findings were higher, except for protein. These differences are most likely due to the case studies reporting the lowest energy and macronutrient intakes at the end of contest preparation, whereas in the current study, we assessed energy and macronutrient intakes at a mean of 8.1 weeks (95% CI 6.8–9.4) out from their competitions. In addition, as was the case in Spendlove et al. [10], the contribution of dietary supplements to dietary intake was not included in the analyses for three of the case studies [6,20,23]. It should also be noted that the contribution of all dietary supplements was only included in dietary analyses for Chappell et al. [21] and Chappell et al. [15], while the contribution of only protein powders was included in the dietary analyses, i.e., energy and protein intake only, for the Halliday et al. [7] case study.

Nutrition recommendations for macronutrient intakes for in-season and off-season competitors have been recently updated and are presented in Table 4 [8,13]. Our study’s competitors fell within those recommendations, except for protein in both seasons, energy during the off-season, and fat during the in-season. The consumption of excess protein could displace carbohydrate intake, thus leading to decreased training performance [29]. Greater protein intake of up to 3.5 g/kg/day, however, has the potential to increase satiety, alleviate hunger, and reduce stress during the in-season [13]. At the very least, it may be advisable for in-season competitors to decrease fat intake to 0.9 g/kg/day and increase carbohydrate intake by a similar amount to help maintain training performance.

Off-season competitors consumed considerably more protein and lower energy intake than the recommendations [8]. This excess protein intake seems to be displacing carbohydrate intake, as it is on the low end of the recommendations. To maximize training performance, recovery, and hypertrophy, it is advised to lower protein intake to at least the upper limit of the recommendation, while concurrently increasing carbohydrate intake over time to meet the carbohydrate recommendations and the necessary energy recommendations to achieve their physique goals [8].

#### 4.1.2. Micronutrients

Micronutrient and other nutrient intakes were similar between in-season and off-season competitors, except in-season competitors’ sugar, saturated fat, monounsaturated fat, polyunsaturated fat, and linoleic acid intakes were lower and vitamin A intake was higher than those of off-season competitors. As noted in Table 5, all micronutrient intakes for in-season and off-season competitors were above the RDA/AI recommendations, which contrasts with a preponderance of the literature spanning from 1989 through 2019. A systematic review examining dietary intake in competitive women bodybuilders noted the following vitamins and minerals were less than the RDA/AI recommendations for in-season competitors: vitamin E, vitamin C, folate, vitamin B12, vitamin B6, calcium, iron, zinc, potassium, and magnesium [10]. Ismaeel et al. [22] also noted the following vitamins and minerals were below 100% of the RDA/AI recommendations: vitamin A, vitamin D, vitamin E, iron, and potassium for in-season competitors. Neither the study by Ismaeel et al. [22] or studies in the systematic review by Spendlove et al. [10] noted the inclusion of dietary supplements in the dietary analyses, except for Newton et al. [18]. Newton et al. [18] discovered that when dietary supplements were included in the dietary analyses, all of the vitamins and minerals for in-season competitors were more than the RDA/AI recommendations, except for potassium (70% of RDA/AI).

Our study did not find any micronutrient values below the RDA/AI when dietary supplements were included in the analyses. However, magnesium and niacin were over the UL for both in-season and off-season competitors. It should be noted, however, that the UL for magnesium only represents intake from medications and dietary supplements, not from food and beverages [66]. Spendlove et al. [10] also indicated that niacin, vitamin B6, iron, and sodium were above the UL only for in-season competitors. Our study concurs with Spendlove et al. [10] for niacin being above the UL for in-season competitors, but not the other micronutrients. Ismaeel et al. [22], however, reported that none of micronutrients were near the UL for in-season competitors. To our knowledge, this is the first study to provide insight into meeting the RDA/AI and exceeding the UL for several micronutrients with the inclusion of dietary supplements in off-season competitors.

Micronutrients that routinely exceed the UL have the potential for adverse effects related to toxicity. Excess intakes of niacin and magnesium most often occur with the consumption of large amounts of dietary supplements [66,67]. The most common side effect of the niacin intake, with a minimum of 30 mg/day, is flushing, mostly in the upper body and face [67]. High doses of magnesium from dietary supplements have the potential to result in nausea, abdominal cramping, and diarrhea [66]. It would be prudent for health practitioners and coaches to inform and educate competitive women bodybuilders on the potential risks of toxicity with routine excess consumption of micronutrients, particularly through their use of dietary supplements.

### 4.2. Dietary Supplement Use

The use of dietary supplements over the past 12 months was reported by 100% of competitive women bodybuilders during the in-season and off-season. In-season and off-season competitors used similar numbers of supplements during peak week and over the past 12 months; however, in-season competitors used more supplements during the in-season, while off-season competitors used more dietary supplements during the off-season. In addition, the number and variety of dietary supplements have increased since the 1980s, as reported in Table 7 and the Supplementary Materials, Table S1. In the systematic review by Spendlove et al. [10], only four of the eight studies reported up to eight dietary supplements used during the in-season, while only one study ascertained two dietary supplements used during the off-season. Although there are limited details on the types and frequency of dietary supplements used in these earlier studies, dietary supplement usage was reported by 100% of in-season competitors, which is congruent with our findings.

More recently, four case studies [6,7,20,23] and two longitudinal studies [15,21] assessed dietary supplement use in in-season competitors; however, to the authors’ knowledge, no recent study has reported dietary supplement use for off-season competitors. Similar to Spendlove et al. [10], these six recent studies reported that 100% of the competitive women bodybuilders used dietary supplements at one time point during their in-season. Although the number of dietary supplements reported in our study for in-season competitors was more than three of the case studies [7,20,23], Tinsley et al. [6] reported a similar number (range = 9–21) of supplements were used during the in-season. Our study and the study by Tinsley et al. [6] may be a more accurate representation of dietary supplement use in competitive women bodybuilders as they are both more recent than the three case studies [7,20,23]. It should be noted that although the number of dietary supplements used in our study is only similar to Tinsley et al. [6], approximately 94% of the types of dietary supplements used by in-season competitors in these case studies were also reported in our study. Only two dietary supplements, i.e., L-tyrosine and ketone supplements, from the Tinsley et al. [6] study were not reported in our study.

Similar to the three case studies [7,20,23], the two UK studies [15,21] reported lower mean dietary supplement intakes than our study. Our study indicated a mean number of dietary supplements used by in-season competitors (mean = 13.0) to be higher compared to the Chappell et al. [21] (mean = 5.4) and Chappell et al. [15] (mean = 8.8) studies. Although the reason for these differences is not completely clear, there may be differences in dietary supplement intake patterns between US and UK in-season competitors. In addition, those two studies only examined natural competitive women bodybuilders, and our study has competitive women bodybuilders who self-reported as both natural, i.e., competitors who do not use performance-enhancing drugs, and enhanced, i.e., competitors who use performance-enhancing drugs, during the in-season, which may affect the number of dietary supplements used.

Although the reported frequency of dietary supplement use for UK in-season competitors differs from those in our study, the types of dietary supplements used in the UK studies are similar (Supplementary Materials, Table S2). Chappell et al. [21] indicated the 12 most common categories of dietary supplements, and the top five in order from most to least frequently used are protein powders, multivitamins, branched-chain amino acids, creatine, and fat burners, while Chappell et al. [15] indicated the top 14 dietary supplement categories, and the top five from most to least frequently used are protein powders, branched-chain amino acids, vitamin C, omega-3, and multivitamins. These two studies, however, only indicated the common types of dietary supplements used during the in-season by UK competitors, whereas our study provides greater detail on the individual dietary supplements used over the entire competition season [the past 12 months] in a large representative sample of US competitive women bodybuilders. The extended time frame assists in examining how use varies over the entire competitive season. Additionally, to our knowledge, dietary supplement use in off-season competitors has been limited to only two case studies since the 1990s [7,20]. These details provide greater insight into the dietary supplement intake patterns of competitive women bodybuilders and the health professionals and coaches working with them.

Although Newton et al. [18] indicated that in-season competitors would have been deficient in several vitamins and minerals without supplementation, the use of dietary supplements is not without the potential for harm. A recent narrative literature review [68] indicated anabolic steroids are often modified for sale as dietary supplements purported to have the same benefits as steroids, and they have the same negative side effects, e.g., cardiovascular morbidity and premature death [69]. O’Dwyer and Vegiraju [35] indicated several classes of dietary supplements that are known to be harmful: weight loss, energy enhancement, sports performance enhancement, and sexual enhancement. Many of these supplements have been reported in the present study to be consumed by competitive women bodybuilders. In a 2013 report from the US Government Accountability Office, 6307 reports of adverse health problems, i.e., serious medical events (53%), hospitalization (29%), serious injury/illness (20%), life-threatening conditions (8%), and death (2%), were received between 2008 to 2011 for dietary supplements [70]. Competitive women bodybuilders could benefit from working with a health professional when considering using dietary supplements.

### 4.3. Diet Quality

Overall diet quality and the HEI-2015 component scores for in-season and off-season competitors are similar, except for a higher total vegetable and added sugar score for in-season competitors than off-season competitors. The Index of Nutritional Quality has been compared with the competitors’ nutrient density for in-season and off-season competitors [26]. Both groups of competitors exceeded the RDA/AI for all nutrients, except calcium, zinc, and iron. A lack of dairy foods, whole grains, and poor variety in protein sources were postulated to be the cause for those low mineral intakes. It should be noted that dietary supplements, except for protein powders, were excluded from their analyses.

Our analyses did not find competitors, regardless of season, to be below the RDA/AI for those three minerals; however, we did find that competitors only achieved about half of the HEI-2015 maximum component points for dairy (in-season = 5.4 vs. off-season = 4.7) and whole grains (in-season = 5.0 vs. off-season = 5.6), regardless of season. These results concur with earlier findings indicating that the consumption of these food groups needs improvement [26]. In-season and off-season competitors could also improve on several other adequacy components to improve their overall diet quality score: total fruit (all forms of fruit, including fruit juice), whole fruit (all forms of fruit, except fruit juice), greens and beans, total vegetables (off-season competitors only), and fatty acids, i.e., more monounsaturated and polyunsaturated fats and less saturated fats.

Although competitive women bodybuilders’ overall dietary quality could be improved, their diet quality is better than the general population of females in the US. Our study discovered the mean HEI-2015 diet quality score to be 69.2 points out of 100 total points, which was higher than the mean score for US females (60 points) from the What We Eat in America, National Health and Nutrition Examination Survey 2017–2018 [71]. Competitive women bodybuilders also have healthier HEI-2015 component scores for total vegetables (4.3 vs. 3.7), whole grains (5.3 vs. 2.7), fatty acids (7.0 vs. 4.2), refined grains (9.2 vs. 6.3), saturated fats (8.6 vs. 4.9), and added sugars (9.6 vs. 6.5) than US females [71]. US females, however, have healthier scores than competitive women bodybuilders for total fruit (3.0 vs. 2.8), whole fruit (4.6 vs. 3.5), dairy (5.7 vs. 5.0), seafood and plant protein (5.0 vs. 4.3), and sodium (4.5 vs. 1.3), while total protein foods (5.0 vs. 5.0) and greens and beans (3.6 vs. 3.7) are essentially the same for both groups [71].

Competitive women bodybuilders could benefit from better diet quality to not only improve nutrient intakes and diet diversity but also improve health outcomes. Hu et al. [72], in a large prospective study examining the incidence of cardiovascular disease, cardiovascular disease mortality, and all-cause mortality across median quintile HEI-2015 scores adjusted for multiple potential confounders in US adults (56% women), found those in the highest quintile of the HEI-2015 (median = 81) had a 16% lower risk of incident cardiovascular disease, 32% lower risk of cardiovascular disease mortality, and 18% lower risk of all-cause mortality than those in the lowest quintile of the HEI-2015 (median = 60). Although competitive women bodybuilders in the present study scored slightly higher diet quality scores than the participants in the Hu et al. [72] study, an improvement in the competitive women bodybuilders’ scores could still provide cardiovascular health benefits. Hu et al. [72] found that for each one standard deviation (8.3 points) increase in the HEI-2015 score, there is a reduced risk of cardiovascular disease by 3–7%, cardiovascular mortality by 8–17%, and all-cause mortality by 4–11% [56]. Similarly, Shan et al. [73], using data from three large prospective cohorts, i.e., Nurses’ Health Study and Nurses’ Health Study II as well as the Health Professionals Follow-up Study, examined the relationship between incident cases of cardiovascular disease, i.e., fatal and nonfatal coronary heart disease and stroke, with median quintile HEI-2015 scores adjusted for multiple potential confounders in US adults. Those participants in the highest quintile (median = 75), compared with those in the lowest quintile (median = 52), had lower incident cases of cardiovascular disease (pooled multivariate-adjusted hazard ratios = 0.83) [73]. Similarly, each 25-point increase in the HEI-2015 score was associated with a 10–20% lower risk of cardiovascular disease (pooled hazard ratio = 0.80) [73]. Competitive women bodybuilders could benefit from improved food choices and meal planning assistance by health professionals and coaches to enhance their diet quality and subsequent health outcomes.

### 4.4. Strengths and Limitations

There are several strengths to this study, but also a few limitations. To the authors’ knowledge, this was one of the largest and most comprehensive dietary intake studies on a nationally representative sample of competitive women bodybuilders, particularly with the inclusion of the contribution of dietary supplements to dietary intake and women in the newer bodybuilder divisions. This was also the first time diet quality had been ascertained in competitive women bodybuilders using the HEI-2015, a valid and reliable tool [30]. In addition, to the authors’ knowledge, this was the first time a validated questionnaire was developed and used to ascertain the sociodemographic, training, competition, and dietary supplement usage characteristics for competitive women bodybuilders. Regardless, it should be noted that the collected data were self-reported and could possibly contain inaccuracies. Moreover, inaccuracies in a participant’s weight could have affected the calculations for energy and macronutrient intakes relative to body weight. In contrast to the general population, bodybuilders are used to regularly weighing themselves [15], and keeping detailed diet and training diaries [25].

Although underreporting is common in dietary studies involving non-bodybuilding populations, it is unknown with bodybuilders [74]. Several techniques, however, were employed to increase the accuracy of the measurement of usual dietary intake in competitive women bodybuilders. Four dietary recalls have been recommended [75], in order to be within 10% of the 365-day average intake for energy, protein, and carbohydrates in females ranging from 20–53 years old. To accurately estimate fat intake, however, they indicated it would require at least six days, and other nutrients require periods ranging from a low of four days for phosphorus to a maximum of thirty-nine days for vitamin A [75]. Given this, a reasonable compromise must be struck between scientific rigor and subject burden; four to five days have been recommended for the collection of dietary intakes [76,77,78,79]. In addition, the automated multiple-pass method, used in the ASA24, improves the accuracy of estimating self-reported dietary intake in lean individuals, e.g., with only 6% underreporting in lean women [80,81]. Using this computer-assisted method of dietary intake analysis, energy intake was underreported by less than 3% compared to the total energy expenditure assessed via doubly labeled water in normal-weight subjects [80]. Also, the ASA24 is a valid and cost-effective tool for collecting dietary intake for research purposes [56]. It should be noted that although underreporting is common when studying the dietary intake of groups, bodybuilders are known to weigh their food and maintain/follow detailed diet plans for long periods of time, often consuming the same types of foods [15,25]. The final two strengths of this study were that the expertise of a dietitian was utilized for the dietary intake and dietary supplement data collection and analyses, and competitive women bodybuilders were used in the design of the questionnaire.

## 5. Conclusions

Competitive women bodybuilding has dramatically increased over the past couple of decades. To obtain their competition-ready physique, diet and supplement intakes are manipulated to extreme levels, raising concern about poor nutrient intakes. In this study we demonstrated that competitive women bodybuilders, whether off-season or in-season, consumed adequate carbohydrates but reported higher fat and protein intakes than recommended. A lower protein intake and higher energy intake, via increased carbohydrate consumption, is recommended for off-season competitors to achieve their physique goals. All competitors met the RDA/AIs for all micronutrients and other nutrients, except for fiber [off-season only] and linoleic acid [in-season only]. Two micronutrients, i.e., niacin and magnesium, were over the UL in all competitors. Competitors should not routinely exceed the UL for micronutrients as that could increase the risk for adverse effects related to toxicity. Future research using a longitudinal design is recommended to assess the potential for micronutrient toxicity and further examine how dietary intake, supplement use, and diet quality changes across seasons, including peak week, competition day, and post-competition in competitive women bodybuilders.

Although competitors’ diets can be routine, lack variety, and be restrictive, their diets are generally able to meet their nutrient needs, with the addition of dietary supplements, and achieve higher diet quality scores than the US population. To reduce the risk of cardiovascular disease, stroke, and overall mortality, however, improvements in the dietary intake for several HEI-2015 components are recommended. In addition, dietary supplement use is ubiquitous in this population, raising the potential for adverse health effects. Competitive women bodybuilders can benefit from nutrition and dietary supplement education to meet the energy and macronutrient recommendations for their training season, while improving their diet quality and use of safe and efficacious dietary supplements. Health professionals and coaches can play a pivotal role in providing this nutrition and dietary supplement education to assist competitive women bodybuilders in achieving their physique goals, lowering their disease risk, and improving their health outcomes.

## Figures and Tables

**Table 1:** Sociodemographic and training characteristics of competitive women bodybuilders.

Characteristic		In-Season (*n* = 112)			Off-Season (*n* = 115)			Totals (*n* = 227)			
			95% CI			95% CI			95% CI		
		M	LB	UB	M	LB	UB	M	LB	UB	*p* *
Age, yr		28.3	27.1	29.4	29.7	28.4	31.1	29.0	28.1	29.9	0.103
Height, cm		163.8	162.8	165.1	164.4	163.1	165.6	164.1	163.3	164.9	0.629
Current Weight, kg		58.6	57.4	59.9	62.6	61.2	63.9	60.6	59.6	61.6	<0.001
Training ^b^											
	Total activity, MET min/wk ^c^	4829.2	4411.6	5246.7	3935.3	3577.5	4293.1	4376.3	4097.8	4654.8	0.001
	Aerobic training, MET min/wk	2658.2	2322.1	2994.3	1880.0	1582.5	2177.5	2264.0	2035.8	2492.1	<0.001
	Resistance training, MET min/wk	1896.2	1709.3	2083.2	1788.1	1597.3	1978.8	1841.4	1708.7	1974.2	0.423
Ethnicity			***n***	**%**		***n***	**%**		***n***	**%**	0.035 ^a^
	Not of Hispanic, Latino, or Spanish origin		103	92.0		95	82.6		198	87.2	
	Mexican, Mexican American, Hispanic, Latino, or Spanish origin		9	8		20	17.4		29	12.8	
Race											0.876
	White		94	83.9		99	86.8		193	85.3	
	Black or African American		6	5.4		6	5.3		12	5.3	
	Asian/Pacific Islander		4	3.6		3	2.6		7	3.0	
	Other races		8	7.2		7	6.1		15	6.7	
Education											0.768
	GED ^d^, high school diploma, or less than 1 year of college credit		6	5.4		9	7.8		15	6.6	
	1 or more years of college credit, no degree		15	13.4		21	18.3		36	15.9	
	Associate’s degree		11	9.8		6	5.2		17	7.5	
	Bachelor’s degree		57	50.9		54	47.0		111	48.9	
	Master’s degree		16	14.3		16	13.9		32	14.1	
	Professional degree beyond a bachelor’s degree		3	2.7		4	3.5		7	3.1	
	Doctorate degree		4	3.6		5	4.3		9	4.0	
Line of work											0.389
	Employed for wages		63	56.3		59	51.3		122	53.7	
	Self-employed		12	10.7		20	17.4		32	14.1	
	Out of work		2	1.8		2	1.7		4	1.8	
	Homemaker		2	1.8		4	3.5		6	2.6	
	Student		9	8.0		12	10.4		21	9.3	
	Retired		1	0.9		1	0.9		2	0.9	
	Employed and self-employed		4	3.6		6	5.2		10	4.4	
	Student and employed, or self-employed, or both		15	13.4		11	9.6		26	11.5	
	Other combinations		4	3.6		-	-		4	1.8	
Household income per month											0.073
	USD 0 to USD 2999		27	24.1		17	14.8		44	19.4	
	USD 3000 to USD 5999		31	27.7		51	44.3		82	36.1	
	USD 6000 to USD 8999		16	14.3		13	11.3		29	12.8	
	USD 9000 to USD 11,999		10	8.9		8	7.0		18	7.9	
	USD 12,000 or more		28	25.0		26	22.6		54	23.8	

Note. M = mean; LB = lower bound of 95% confidence interval; UB = upper bound of 95% confidence interval; *p* < 0.05 between in-season and off-season competitors; * reported *p* values are unadjusted; ^a^ not significant after Benjamini and Hochberg multiple comparison testing; ^b^ these are the exercises performed only for bodybuilding training and do not include other activities for leisure or work; ^c^ metabolic equivalents in minutes per week; ^d^ general educational development.

**Table 2:** Competition characteristics.

Characteristic			In-Season (*n* = 112)			Off-Season (*n* = 115)			Totals (*n* = 227)			
			*n*	%		*n*	%		*n*	%		*p* *
Competitor division												0.244
		Bikini	82	73.2		71	61.7		153	67.4		
		Bodybuilder	1	0.9		-	-		1	0.4		
		Figure	16	14.3		24	20.9		40	17.6		
		Fitness	2	1.8		7	6.1		9	4		
		Physique	4	3.6		6	5.2		10	4.4		
		Wellness	7	6.3		7	6.1		14	6.2		
Competitor status												0.571
		Amateur	101	90.2		101	87.8		202	89		
		Professional	11	9.8		14	12.2		25	11		
Never competed			28	25		32	27.8		60	26.4		0.629
Most recent top competition placing												
	Organization name											0.632
		NPC	70	83.3		70	84.3		140	83.8		
		IFBB	3	3.6		6	7.2		9	5.4		
		WNBF	3	3.6		3	3.6		6	3.6		
		NANBF	2	2.4		-	-		2	1.2		
		OCB	3	3.6		3	3.6		6	3.6		
		Other organization	3	3.6		1	1.2		4	2.4		
	Year											0.276
		2011–2017	9	10.7		10	12.0		19	11.4		
		2018	7	8.3		15	18.1		22	13.2		
		2019	43	51.2		38	45.8		81	48.5		
		2020	25	29.8		20	24.1		45	26.9		
	Placing											0.596
		1	27	31.8		23	30.3		50	31.1		
		2	16	18.8		20	26.3		36	22.4		
		3	17	20		9	11.8		26	16.1		
		4	7	8.2		10	13.2		17	10.6		
		5	4	4.7		3	3.9		7	4.3		
		6–15	9	10.6		9	11.8		18	11.2		
		Did not place	5	5.9		2	2.6		7	4.3		
	Type											0.339
		Amateur	80	95.2		76	91.6		156	93.4		
		Professional	4	4.8		7	8.4		11	6.6		
	Professional status obtained?											0.695
		Yes	9	11.3		7	9.3		16	10.3		
		No	71	88.8		68	90.7		139	89.7		
Self-reported as a “natural” athlete												0.774
		Yes	92	82.1		96	83.5		188	82.8		
		No	20	17.9		19	16.5		39	17.2		
**Characteristic**			**In-Season (*n* = 112)**			**Off-Season (*n* = 115)**			**Totals (*n* = 227)**			
				**95% CI**			**95% CI**			**95% CI**		
			**M**	**LB**	**UB**	**M**	**LB**	**UB**	**M**	**LB**	**UB**	*p* *
Most recent competition weight, kg			54.2	53.0	55.4	54.9	53.7	56.1	54.6	53.7	55.4	0.400
Lowest off-season weight since last competition, kg			58.0	56.6	59.3	58.2	56.7	59.6	58.1	57.1	59.0	0.830
Highest off-season weight since last competition, kg			64.5	62.8	66.1	66.3	64.6	67.9	65.4	64.2	66.5	0.133
Total number of years competing			2.2	1.8	2.6	2.3	1.8	2.7	2.2	1.9	2.5	0.878
Total number of competitions			3.0	2.4	3.6	3.7	2.9	4.5	3.4	2.9	3.9	0.181
Time to prepare for last competition, wks			18.8	17.4	20.1	21.0	19.5	22.6	19.9	18.8	20.9	0.032 ^a^
Time since last competition, wks			50.4	40.3	60.4	50.3	41.5	59.2	50.4	43.7	57.0	0.961

Note. M = mean; LB = lower bound of 95% confidence interval; UB = upper bound of 95% confidence interval; * reported *p* values are unadjusted; ^a^ not significant after Benjamini and Hochberg multiple comparison testing.

**Table 3:** Energy and macronutrient intakes.

Nutrient		In-Season (*n* = 112)			Off-Season (*n* = 115)			Totals (*n* = 227)			
			95% CI			95% CI			95% CI		
		M	LB	UB	M	LB	UB	M	LB	UB	*p* *
	Energy (kcal)	1779.8	1689.1	1870.4	2032.9	1940.6	2125.1	1906.3	1841.7	1970.9	<0.001
	Protein (g)	155.5	148.5	162.5	141.2	134.1	148.3	148.4	143.4	153.4	0.005
	Carbohydrate (g)	156.0	143.5	168.5	199.1	186.3	211.8	177.5	168.6	186.5	<0.001
	Fat (g)	60.1	55.6	64.6	70.9	66.3	75.5	65.5	62.3	68.7	<0.001

Note. M = mean; LB = lower bound of 95% confidence interval; UB = upper bound of 95% confidence interval; *p* < 0.05 between in-season and off-season competitors; * reported *p* values are unadjusted.

**Table 4:** Relative energy and macronutrient intakes by season compared to nutrition recommendations.

Nutrient	In-Season (*n* = 112)					Off-Season (*n* = 115)					
	Physique Athlete		95% CI		% Recommendation Met ^a^	Physique Athlete		95% CI		% Recommendation Met ^a^	
	Recommendations [13]	M	LB	UB		Recommendations [8]	M	LB	UB		*p* *
Energy (kcal)	-	33.3	31.6	34.9	-	42–48 kcal/kg	38.0	36.3	39.7	90.4%	<0.001
Protein (g)	1.8–2.7 g/kg	2.9	2.8	3.0	107.6%	1.5–1.9 g/kg	2.6	2.5	2.8	138.9%	0.005
Carbohydrate (g)	2–5 g/kg	2.9	2.7	3.1	100.0%	3–5 g/kg	3.7	3.5	4.0	100.0%	<0.001
Fat (g)	0.4–0.9 g/kg	1.1	1.0	1.2	121.6%	0.5–1.5 g/kg	1.3	1.2	1.4	100.0%	0.001

Note. *p* < 0.05 between in-season and off-season competitors: * reported *p* values are unadjusted; M = mean; LB = lower bound of 95% confidence interval; UB = upper bound of 95% confidence interval. ^a^ The percent recommendation met by season was determined through dividing the mean intake for each season by the upper end of the recommendation range multiplied by 100. The upper end of the recommendation was selected to determine the percent recommendation value, because this is the maximum level of intake recommended that may not potentially negatively affect obtaining the recommended intakes for the other macronutrients. If the mean intake for each season fell within the recommendation range, then the percent recommendation met was given a value of 100%.

**Table 5:** Micronutrient and other nutrient intakes by season.

Nutrient	In-Season (*n* = 112)			Off-Season (*n* = 115)			Totals (*n* = 227)			
		95% CI			95% CI			95% CI		
	M	LB	UB	M	LB	UB	M	LB	UB	*p* *
Caffeine (mg)	136.3	110.9	161.8	118.2	92.3	144.1	127.3	109.1	145.4	0.327
Sugars (g)	48.0	43.1	52.9	62.0	57.0	66.9	55.0	51.5	58.5	<0.001
Fiber (g)	25.2	23.1	27.2	24.4	22.3	26.5	24.8	23.3	26.3	0.611
Calcium (mg)	1265.8	1160.4	1371.2	1196.0	1088.7	1303.3	1230.9	1155.8	1306.0	0.363
Iron (mg)	18.9	17.0	20.8	22.0	20.1	24.0	20.5	19.1	21.8	0.025 ^a^
Magnesium (mg)	537.4	497.1	577.7	515.4	474.4	556.4	526.4	497.7	555.1	0.453
Phosphorus (mg)	1899.7	1801.9	1997.4	1738.3	1638.9	1837.7	1819.0	1749.3	1888.6	0.023 ^a^
Potassium (mg)	3654.9	3443.4	3866.4	3365.2	3150.0	3580.4	3510.1	3359.3	3660.8	0.060
Sodium (mg)	4033.8	3800.8	4266.8	4054.8	3817.7	4291.9	4044.3	3878.3	4210.3	0.902
Zinc (mg)	17.9	15.8	20.1	21.0	18.8	23.2	19.5	17.9	21.0	0.046 ^a^
Copper (mg)	2.0	1.8	2.2	2.0	1.9	2.2	2.0	1.9	2.1	0.631
Selenium (µg)	196.5	181.4	211.6	186.2	170.8	201.6	191.4	180.6	202.1	0.347
Vitamin C (mg)	252.5	202.2	302.9	182.3	131.0	233.5	217.4	181.5	253.3	0.055
Thiamin (mg)	5.0	2.4	7.5	5.3	2.7	7.9	5.1	3.3	6.9	0.854
Riboflavin (mg)	6.3	3.8	8.7	7.2	4.7	9.7	6.7	5.0	8.5	0.597
Niacin (mg)	51.8	46.7	57.0	48.3	43.1	53.5	50.1	46.4	53.7	0.345
Vitamin B_6_ (mg)	7.7	5.2	10.3	7.2	4.6	9.8	7.5	5.6	9.3	0.757
Folate (µg)	582.9	515.2	650.5	602.6	533.7	671.4	592.7	544.5	640.9	0.689
Vitamin B_12_ (µg)	39.1	16.7	61.5	28.9	6.1	51.7	34.0	18.0	49.9	0.531
Vitamin A (µg_RAE)	1544.7	1355.0	1734.5	1090.8	897.7	1283.9	1317.7	1182.5	1453.0	0.001
Vitamin E (mg)	17.7	14.7	20.8	15.8	12.6	18.9	16.7	14.6	18.9	0.373
Vitamin K (µg)	272.3	212.0	332.7	259.4	198.0	320.8	265.9	222.9	308.9	0.769
Cholesterol (mg)	429.2	387.4	471.0	395.0	352.5	437.6	412.1	382.3	441.9	0.262
Saturated fat (g)	16.1	14.5	17.7	20.2	18.6	21.8	18.1	17.0	19.3	<0.001
Monounsaturated fat (g)	21.6	19.8	23.5	25.3	23.4	27.2	23.5	22.1	24.8	0.007
Polyunsaturated fat (g)	13.1	11.9	14.3	15.5	14.4	16.7	14.3	13.5	15.2	0.004
Linoleic acid (g)	10.6	9.6	11.7	13.3	12.3	14.3	12.0	11.2	12.7	<0.001
α-Linolenic acid (g)	1.3	1.1	1.4	1.3	1.2	1.4	1.3	1.2	1.4	0.662
Vitamin D (µg)	19.1	14.3	23.9	17.3	12.4	22.2	18.2	14.8	21.6	0.598
Choline (mg)	520.1	485.5	554.7	465.0	429.8	500.2	492.5	467.9	517.2	0.029 ^a^

Note. M = mean; LB = lower bound of 95% confidence interval; UB = upper bound of 95% confidence interval; *p* < 0.05 between in-season and off-season competitors; * reported *p* values are unadjusted; ^a^ not significant after Benjamini and Hochberg multiple comparison testing.

**Table 6:** Dietary supplement use across seasons during the past 12 months by competitive women bodybuilders currently in-season and off-season.

Period Reported for	Period Data Collected									
	In-Season Competitors (*n* = 112)			Off-Season Competitors (*n* = 115)			Totals (*n* = 227)			
		95% CI			95% CI			95% CI		
	M	LB	UB	M	LB	UB	M	LB	UB	*p*
Off-season	8.8 ^b^	7.9	9.7	10.3 ^b^	9.4	11.2	9.5 ^b^	8.9	10.2	0.034
In-season	11.2 ^a^	10.1	12.2	9.5 ^b^	8.4	10.5	10.3 ^b^	9.6	11.0	0.030
Peak week	3.2	2.7	3.8	3.0	2.5	3.5	3.1	2.7	3.5	0.567
Totals	13.0	11.9	14.2	13.1	11.9	14.2	13.1	12.3	13.9	0.955

Note. M = mean; LB = lower bound of 95% confidence interval; UB = upper bound of 95% confidence interval; *p* < 0.05 between in-season and off-season competitors. Reported *p* values are adjusted for multiple comparisons (Sidak). ^a^ Significantly greater than off-season dietary supplement use and peak week dietary supplement use (*p* < 0.05) according to Sidak’s test; ^b^ significantly greater than peak week dietary supplement use (*p* < 0.05) according to Sidak’s test.

**Table 7:** Diet quality by season.

Nutrient			In-Season (*n* = 112)			Off-Season (*n* = 115)			Totals (*n* = 227)			
		Score Range ^b^		95% CI			95% CI			95% CI		
			M	LB	UB	M	LB	UB	M	LB	UB	*p*
HEI-2015 Total score		0–100 ^c^	70.2	66.2	74.1	68.2	64.3	72.1	69.2	66.4	71.9	0.480
HEI-2015 components scores												
	Adequacy—dietary components that should be consumed in sufficient quantities for overall good health.											
	Total vegetables	0–5	4.7	4.3	5.1	3.9	3.5	4.3	4.3	4.0	4.6	0.003
	Greens and beans	0–5	3.8	3.0	4.7	3.7	2.8	4.5	3.7	3.2	4.3	0.750
	Total fruit	0–5	2.5	1.8	3.3	3.0	2.2	3.7	2.8	2.2	3.3	0.375
	Whole fruit	0–5	3.2	2.4	4.0	3.7	2.9	4.5	3.5	2.9	4.0	0.353
	Whole grains	0–10	5.0	3.7	6.3	5.6	4.4	6.9	5.3	4.4	6.2	0.484
	Dairy	0–10	5.4	4.1	6.6	4.7	3.4	5.9	5.0	4.1	5.9	0.424
	Total protein foods ^d^	0–5	5.0	5.0	5.0	5.0	5.0	5.0	5.0	5.0	5.0	-
	Seafood and plant protein	0–5	4.8	4.2	5.3	3.8	3.3	4.4	4.3	3.9	4.7	0.017 ^a^
	Fatty acids	0–10	7.2	6.0	8.5	6.7	5.4	7.9	7.0	6.1	7.8	0.519
	Moderation—dietary components that should be consumed in limited quantities.											
	Sodium	0–10	0.8	0.1	1.5	1.8	1.1	2.5	1.3	0.8	1.8	0.047 ^a^
	Refined grains	0–10	9.8	9.0	10.6	8.6	7.9	9.4	9.2	8.7	9.7	0.030
	Saturated fats	0–10	8.7	7.8	9.6	8.6	7.7	9.5	8.6	8.0	9.3	0.899
	Added sugars	0–10	9.8	9.6	9.9	9.5	9.3	9.6	9.6	9.5	9.7	0.005

Note. M = mean; LB = lower bound of 95% confidence interval; UB = upper bound of 95% confidence interval; *p* < 0.05 between in-season and off-season competitors; reported *p* values are unadjusted. ^a^ Not significant after Benjamini and Hochberg multiple comparison testing. ^b^ The higher the score for the first eight HEI components, the greater the consumption. The higher the score for the last four HEI components, the lower the consumption. ^c^ The higher the total HEI score, the better the diet quality. ^d^ After 90% winsorization, all participations were at a value of five in total protein foods, as only four participants had a value less than five prior to winsorization.

## Data Availability

The data presented in this study are available on request from the corresponding author. The data are not publicly available due to the nature of participant confidentiality.

## Supplementary Materials

The following supporting information can be downloaded at https://www.mdpi.com/article/10.3390/sports11080158/s1. Table S1: Micronutrient and other nutrient intakes by season compared to the Recommended Dietary Allowance/Adequate Intake (RDA/AI) values; Table S2: Dietary supplement use by frequency and percentage compared across seasons; Table S3: Dietary supplement use frequency, percentages, and position among seasons; Table S4: Number of dietary supplements used in the past 12 months by cumulative frequency and percent.
